# Long-term dynamic compression enhancement TGF-β3-induced chondrogenesis in bovine stem cells: a gene expression analysis

**DOI:** 10.1186/s12863-021-00967-2

**Published:** 2021-03-20

**Authors:** Jishizhan Chen, Lidan Chen, Jia Hua, Wenhui Song

**Affiliations:** 1 UCL Centre for Biomaterials in Surgical Reconstruction and Regeneration, Division of Surgery & Interventional Science, University College London, London, NW3 2PF UK; 2 Centre of Maxillofacial Surgery and Digital Plastic Surgery, Plastic Surgery Hospital, Chinese Academy of Medical Sciences and Peking Union Medical College, Beijing, 100144 People’s Republic of China; 3 UCL Institute of Orthopaedics and Musculoskeletal Science, Division of Surgery & Interventional Science, University College London, Stanmore, London, HA7 4AP UK; 4 The Griffin Institute (Northwick Park Institute for Medical Research), Harrow, London, HA1 3UJ UK; 5 Faculty of Science and Technology, Middlesex University, London, NW4 4BT UK

**Keywords:** Bioinformatics, Chondrogenesis, Enrichment analysis, Mechanical stimulation, Mesenchymal stem cells

## Abstract

**Background:**

Bioengineering has demonstrated the potential of utilising mesenchymal stem cells (MSCs), growth factors, and mechanical stimuli to treat cartilage defects. However, the underlying genes and pathways are largely unclear. This is the first study on screening and identifying the hub genes involved in mechanically enhanced chondrogenesis and their potential molecular mechanisms.

**Methods:**

The datasets were downloaded from the Gene Expression Omnibus (GEO) database and contain six transforming growth factor-beta-3 (TGF-β3) induced bovine bone marrow-derived MSCs specimens and six TGF-β3/dynamic-compression-induced specimens at day 42. Screening differentially expressed genes (DEGs) was performed and then analysed via bioinformatics methods. The Database for Annotation, Visualisation, and Integrated Discovery (DAVID) online analysis was utilised to obtain the Gene Ontology (GO) and Kyoto Encyclopaedia of Genes and Genomes (KEGG) pathway enrichment. The protein-protein interaction (PPI) network of the DEGs was constructed based on data from the STRING database and visualised through the Cytoscape software. The functional modules were extracted from the PPI network for further analysis.

**Results:**

The top 10 hub genes ranked by their connection degrees were IL6, UBE2C, TOP2A, MCM4, PLK2, SMC2, BMP2, LMO7, TRIM36, and MAPK8. Multiple signalling pathways (including the PI3K-Akt signalling pathway, the toll-like receptor signalling pathway, the TNF signalling pathway, and the MAPK pathway) may impact the sensation, transduction, and reaction of external mechanical stimuli.

**Conclusions:**

This study provides a theoretical finding showing that gene UBE2C, IL6, and MAPK8, and multiple signalling pathways may play pivotal roles in dynamic compression-enhanced chondrogenesis.

## Background

Cartilage has little self-renewable ability due to its instinctive physiologies [1, 2], which include an avascular, aneural and non-lymphatic system [3], low cellularity in adult tissue, and a dense hydrated extracellular matrix, hampering resident chondrocytes or progenitor cells migration to the defect site to secrete a reparative matrix [2]. Mesenchymal stem cells (MSCs) are promising cell sources for osteochondral engineering. Numerous studies have demonstrated successful induction of chondrogenesis in various biomaterials. This strategy shows remarkable potential in repairing cartilage defects caused by osteoarthritis and athletic injuries [4, 5]. The most commonly used chondrogenic medium contains the TGF-β superfamily, which is a crucial mediator of MSCs chondrogenesis. Literature shows that TGF-β has proven a success in inducing chondrogenesis in vitro [6, 7]. However, TGF-β-induced chondrocytes alone were then witnessed a hypertrophic phenotype [8], which is not an ideal cell phenotype. Thus, inhibiting hypertrophy during the chondrogenic process in vitro and maintaining a stable cartilaginous phenotype need to be overcome.

Inspired by the physiology of native articular cartilage subjected to the dynamic joint environment, mainly under compression and shearing conditions [9], the significance of biomechanical stimuli has been well-established in the case of cartilage. Previous studies have shown that the ligand-integrin-cytoskeleton complex is the major mechanosensing component of the cell. The dynamic load and integrin activate the focal adhesion kinase (FAK) and mitogen-activated protein kinase (MAPK) pathways, increase the intracellular calcium, and induce further cell processes [10, 11]. Additionally, there are other pathways that do not rely on calcium. Dynamic compression is the most highly used physical condition to promote chondrogenesis [12]. Dynamic compression has been proven not only to enhance the efficiency of growth factors, but also play an important role in maintaining chondrocytes phenotypes and inhibiting hypertrophy. Despite increasing research on the impact of mechanical stimuli on chondrogenesis, there is no comprehensive understanding of underlying genes, while signal pathways remain elusive. Hence, in order to develop an optimal chondrogenic differentiation strategy, there is a pressing need to identify the key genes and signal pathways involved.

Microarray technology provides a powerful tool for exploring the gene regulation pattern and molecular mechanisms involved in mechanical-enhanced chondrogenesis. It enables to investigate thousands of gene expression patterns [13]. The microarray data can be uploaded and shared through open-source databases such as the Gene Expression Omnibus (GEO) database [14]. Huang et al. [15] provided the first study of how long-term (21 days) dynamic compression affected chondrogenesis. They briefly displayed a preliminary microarray screen for the genome expression profiles with chondrogenic induction and long-term dynamic compression. With limited data currently available on this topic, this study was conducted based on selected Huang’s data on gene expression patterns affected by dynamic compression after a sustained TGF-β3 chondrogenic induction of MSCs, and further analyses shed more in-depth understanding of the underlying mechanisms. Datasets were downloaded containing genes expression data between TGF-β3-induced and TGF-β3/dynamic-compression-induced chondrogenesis of bovine MSCs from the GEO. Differentially expressed genes (DEGs) were screened, and Gene Ontology (GO), Kyoto Encyclopaedia of Genes and Genomes (KEGG), and protein-protein interaction (PPI) network analyses were performed to explore the hub genes and key modules involved. To summarise, 236 DEGs and 10 hub genes were identified, which may be key candidates for responding to dynamic compression during chondrogenic differentiation of MSCs.

## Results

### Data pre-processing and identification of DEGs

**Fig. 1:**
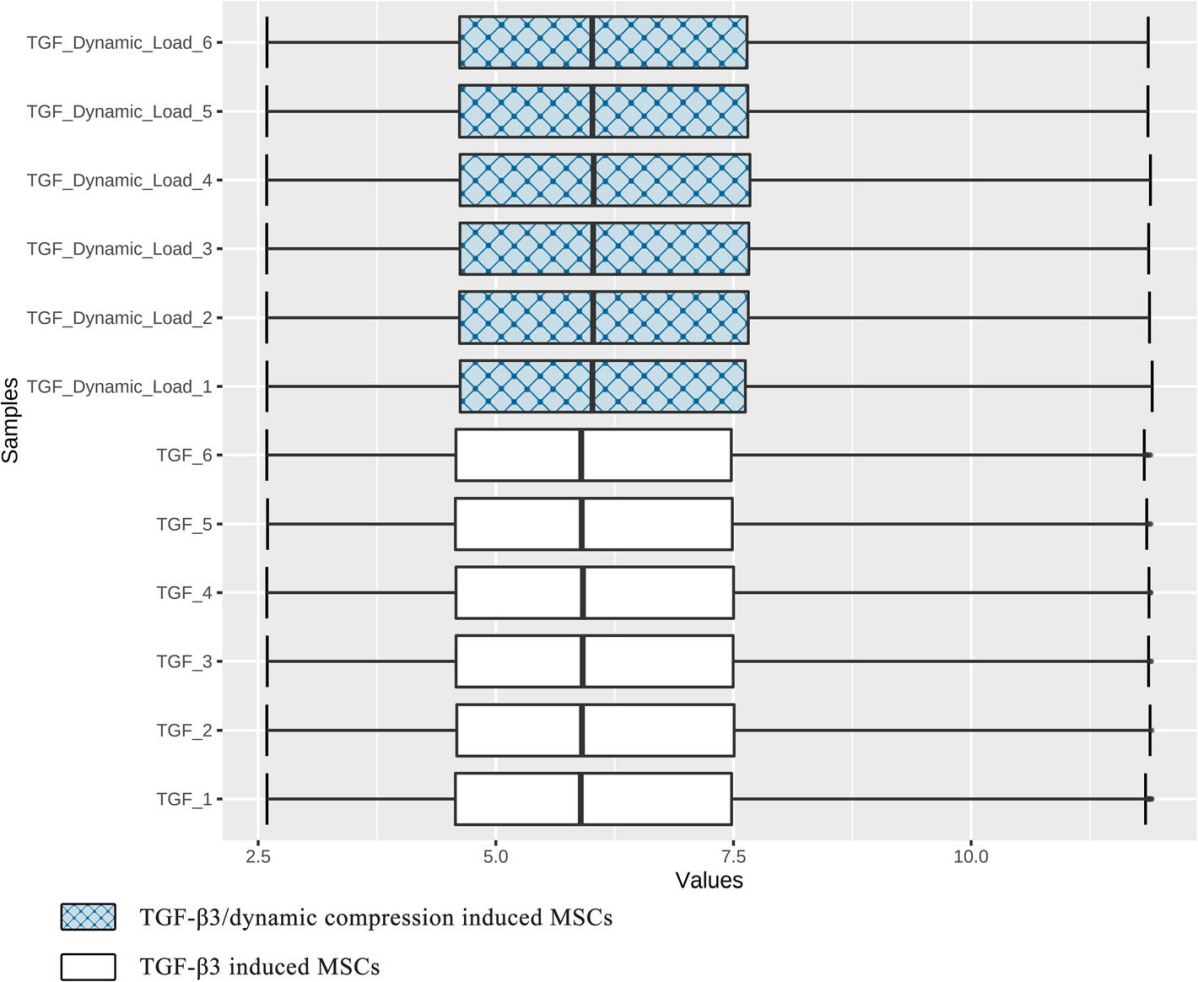
Box-plot of normalised data. Black lines in the boxes represent medians

**Fig. 2:**
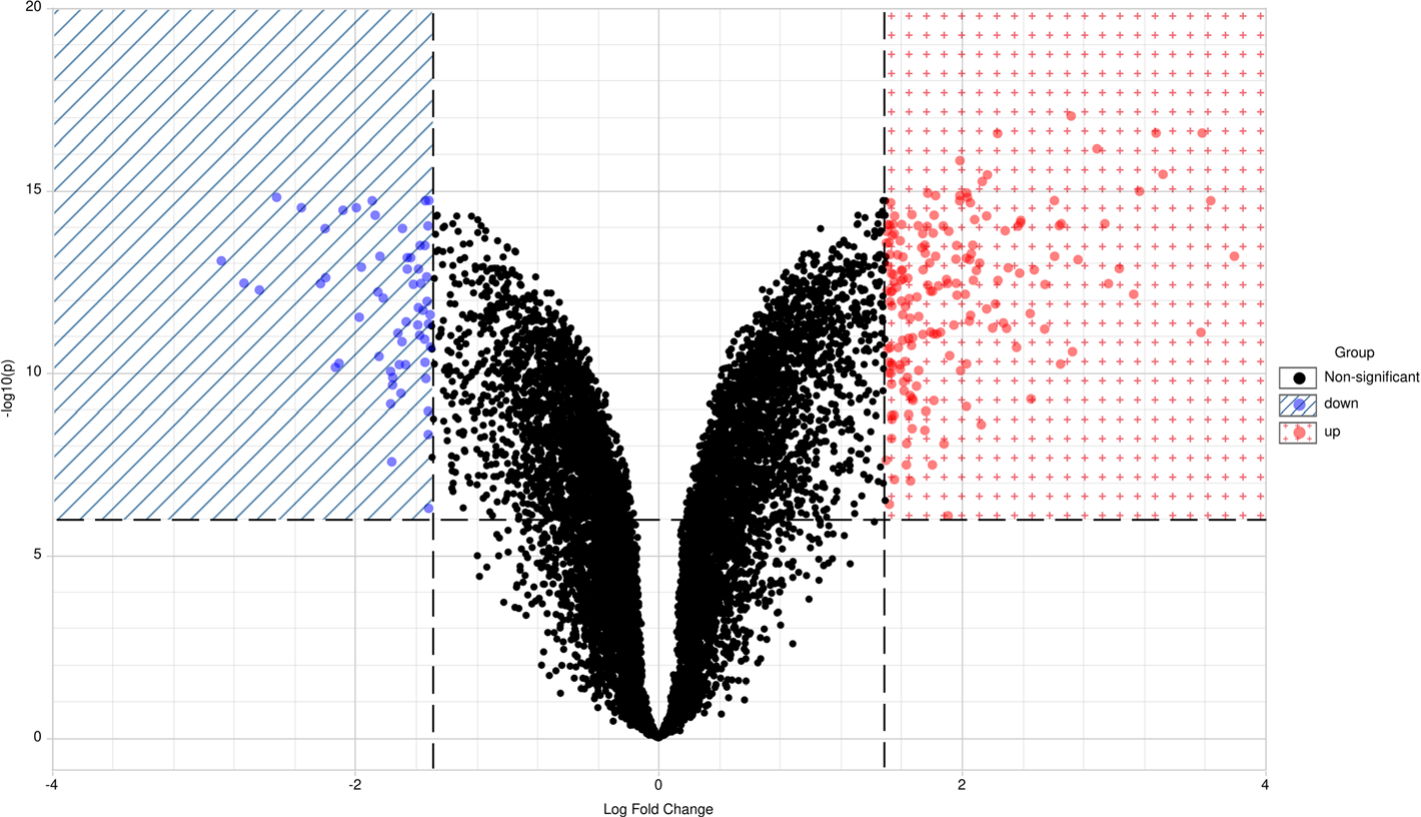
Volcano plot of all genes detected in the microarray. Each dot represents a gene. Dashed lines divide areas of down- and up-regulated genes. The X-axis is log2-base fold change, and Y-axis is −log10-base adjusted *P*-value

**Fig. 3:**
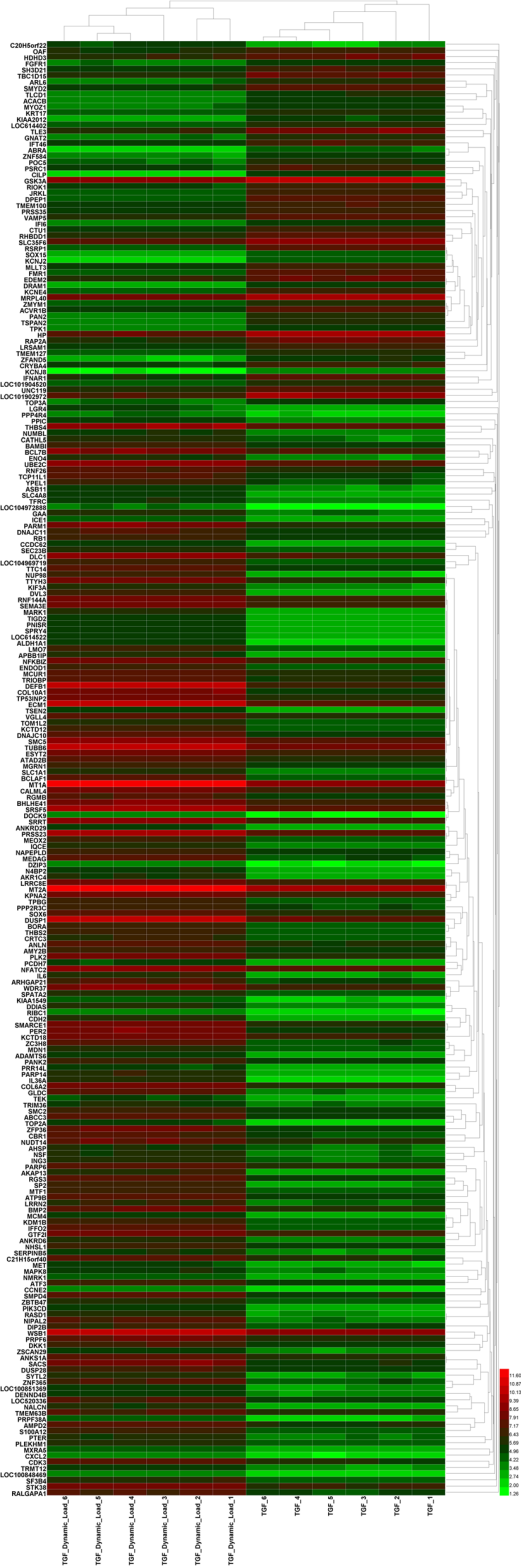
Cluster heatmap demonstrates hierarchical clustering analysis results according to DEGs. Each row represents a DEG, and each column represents a sample. The colour displays the relative gene expression level. Green indicates lower values in gene expression, and red indicates higher values

Figure 1 displays the gene expression data of two groups containing 12 samples after normalisation. Medians show good alignment, indicating a high data quality after normalisation and suitability for the following analyses. The Volcano plot (Fig. 2) demonstrates the differential expression status of all detected genes highlighting DEGs beyond the set cut-off criterion. A total of 236 DEGs were obtained, of which 178 (75.42%) were up-regulated genes, and 58 (24.58%) were down-regulated genes in TGF-β3/dynamic-compression-induced MSCs compared to TGF-β3-induced MSCs. The cluster heatmap of DGEs is displayed in Fig. 3. The Euclidean distance was adopted to cluster the genes and produce the dendrograms. The red and green colours distinguish relatively higher or lower gene expression in each sample. Significant differences in DEGs expression patterns can be observed between these two groups (with and without dynamic compressive stimulation), indicating that the DEGs are reliable and eligible for the following analyses. The top 10 most significantly up-regulated and down-regulated genes are shown in Table 1.

**Table 1:** The top 10 most significantly up-regulated and down-regulated DEGs

Up-regulated DEGs	Log2FC	P-value	Down-regulated DEGs	Log2FC	P-value
**ALDH1A1**	2.7201	9.43 × 10^−18^	**FMR1**	−2.5168	1.55 × 10^−15^
**COL10A1**	3.2805	2.76 × 10^−17^	**SOX15**	−1.5129	1.90 × 10^−15^
**DEFB1**	3.5844	2.76 × 10^−17^	**PAN2**	−1.8864	1.93 × 10^−15^
**LOC614522**	2.2359	2.84 × 10^−17^	**MLLT3**	−1.5365	1.93 × 10^−15^
**APBB1IP**	2.8907	7.23 × 10^−17^	**KCNJ2**	−2.3538	2.99 × 10^−15^
**TOM1L2**	1.9842	1.49 × 10^−16^	**DRAM1**	−1.9913	2.99 × 10^−15^
**TIGD2**	1.9875	1.53 × 10^−16^	**KCNE4**	−2.079	3.51 × 10^−15^
**PER2**	3.3263	3.64 × 10^−16^	**ACVR1B**	−1.8672	4.81 × 10^−15^
**ENDOD1**	2.1683	3.75 × 10^−16^	**ZMYM1**	−1.5191	9.43 × 10^−15^
**TSEN2**	2.1345	5.71 × 10^−16^	**SLC35F6**	−1.6873	1.11 × 10^− 14^

### GO and pathway enrichment analyses

**Fig. 4:**
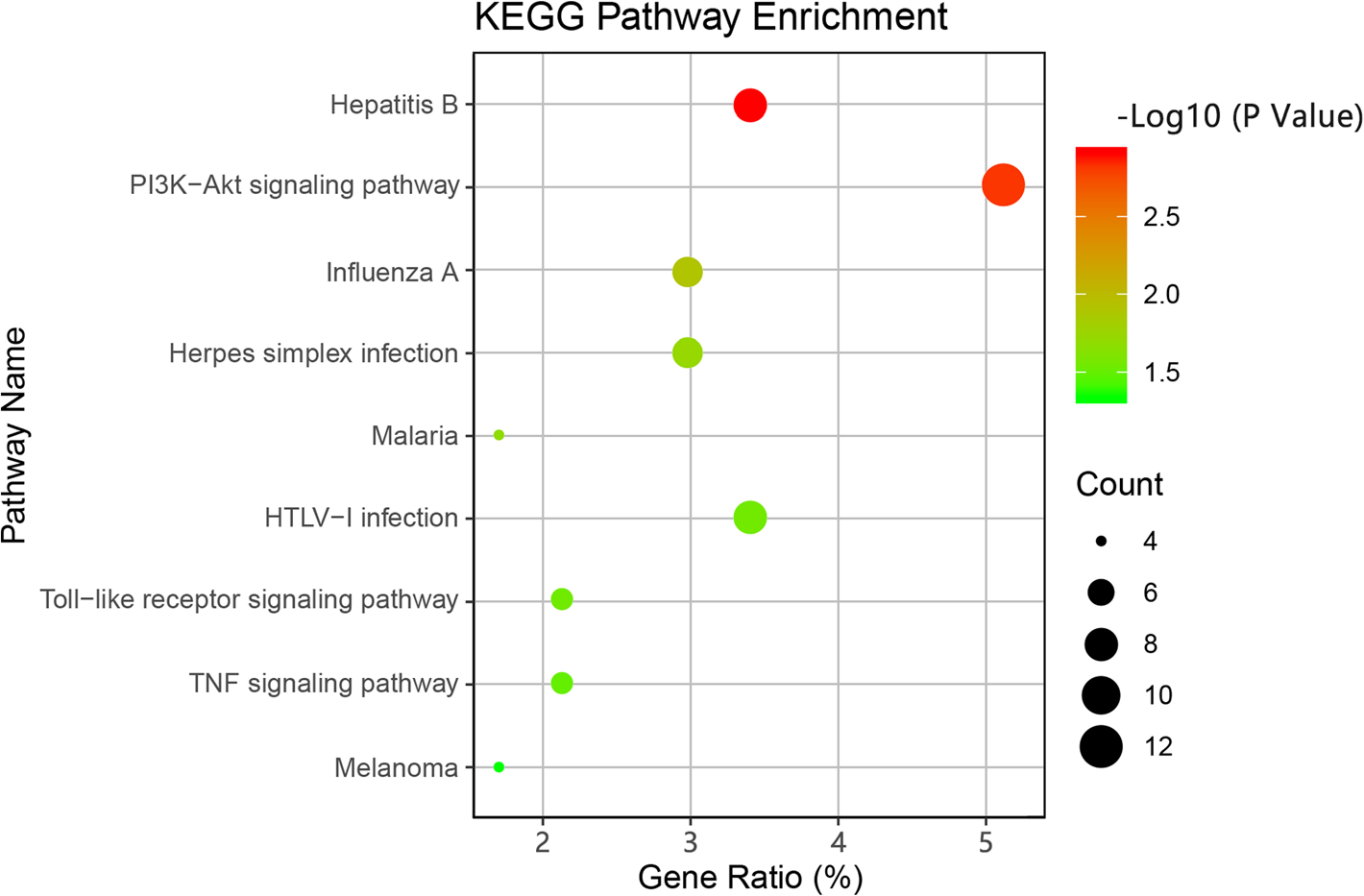
KEGG pathway enrichment analysis. The gradual colour stands for −log10-base adjusted P-value, red indicates a higher adjusted P-value, and green indicates a lower adjusted P-value. Dots size stands for gene count number. The X-axis represents the gene percentage ratio, and the Y-axis lays out pathway names

GO enrichment and KEGG pathway enrichment analyses were performed to identify the biological function of DEGs. In GO terms, a negative regulation of angiogenesis, in utero embryonic development, and inflammatory responses provided the most significant enrichment in the biological process. The most significant enrichment in the cellular component was created through the cytoplasm, transcription elongation factor complex, and cortical actin cytoskeleton. Haemoglobin binding and ATP binding represented the most significant enrichment in the molecular function. A full list of enriched GO terms is shown in Table 2. In the KEGG pathway enrichment analysis, after screening and removing obviously irrelevant disease clusters, the PI3K-Akt signalling pathway, the toll-like receptor signalling pathway, and the TNF signalling pathway were remarkably enriched in dynamic compression-enhanced chondrogenesis (see Fig. 4 and Table 3).

**Table 2:** Significantly enriched GO terms of DEGs

Category	GO ID	Description	Gene Count	P-value
BP	GO:0016525	negative regulation of angiogenesis	5	2.82 × 10^−3^
BP	GO:0001701	in utero embryonic development	7	1.37 × 10^−2^
BP	GO:0097009	energy homeostasis	3	1.42 × 10^−2^
BP	GO:0006954	inflammatory response	8	1.71 × 10^− 2^
BP	GO:0090023	positive regulation of neutrophil chemotaxis	3	2.24 × 10^− 2^
BP	GO:0009611	response to wounding	3	3.75 × 10^−2^
BP	GO:0010718	positive regulation of epithelial to mesenchymal transition	3	4.61 × 10^−2^
CC	GO:0005737	cytoplasm	52	1.47 × 10^−2^
CC	GO:0008023	transcription elongation factor complex	3	2.02 × 10^−2^
CC	GO:0035363	histone locus body	2	4.83 × 10^−2^
CC	GO:0030864	cortical actin cytoskeleton	3	4.91 × 10^−2^
MF	GO:0030492	haemoglobin binding	2	2.50 × 10^− 2^
MF	GO:0005524	ATP binding	24	3.37 × 10^−2^

**Table 3:** Signalling pathway enrichment analysis of DEGs

KEGG ID	Description	Gene Count	P-value	Gene list
bta05161	Hepatitis B	8	1.24 × 10^− 3^	CCNE2, IL6, PIK3CD, MAPK8, RB1, NFATC2, IFNAR1
bta04151	PI3K-Akt signalling pathway	12	1.50 × 10^− 3^	CCNE2, FGFR1, IL6, TEK, PIK3CD, MET, COL6A2, THBS2, PPP2R3C, THBS4, IFNAR1
bta05164	Influenza A	7	1.24 × 10^−2^	IL6, NUP98, PIK3CD, MAPK8, KPNA2, IFNAR1
bta05168	Herpes simplex infection	7	1.88 × 10^− 2^	SRSF5, IL6, GTF2I, PER2, MAPK8, IFNAR1
bta05144	Malaria	4	2.09 × 10^− 2^	IL6, MET, THBS2, THBS4
bta05166	HTLV-I infection	8	2.86 × 10^− 2^	ZFP36, CRTC3, DVL3, IL6, ATF3, PIK3CD, RB1, NFATC2
bta04620	Toll-like receptor signalling pathway	5	2.92 × 10^− 2^	IL6, PIK3CD, MAPK8, IFNAR1
bta04668	TNF signalling pathway	5	3.19 × 10^−2^	IL6, CXCL2, PIK3CD, MAPK8
bta05218	Melanoma	4	4.58 × 10^−2^	FGFR1, PIK3CD, MET, RB1

### PPI network construction

**Fig. 5:**
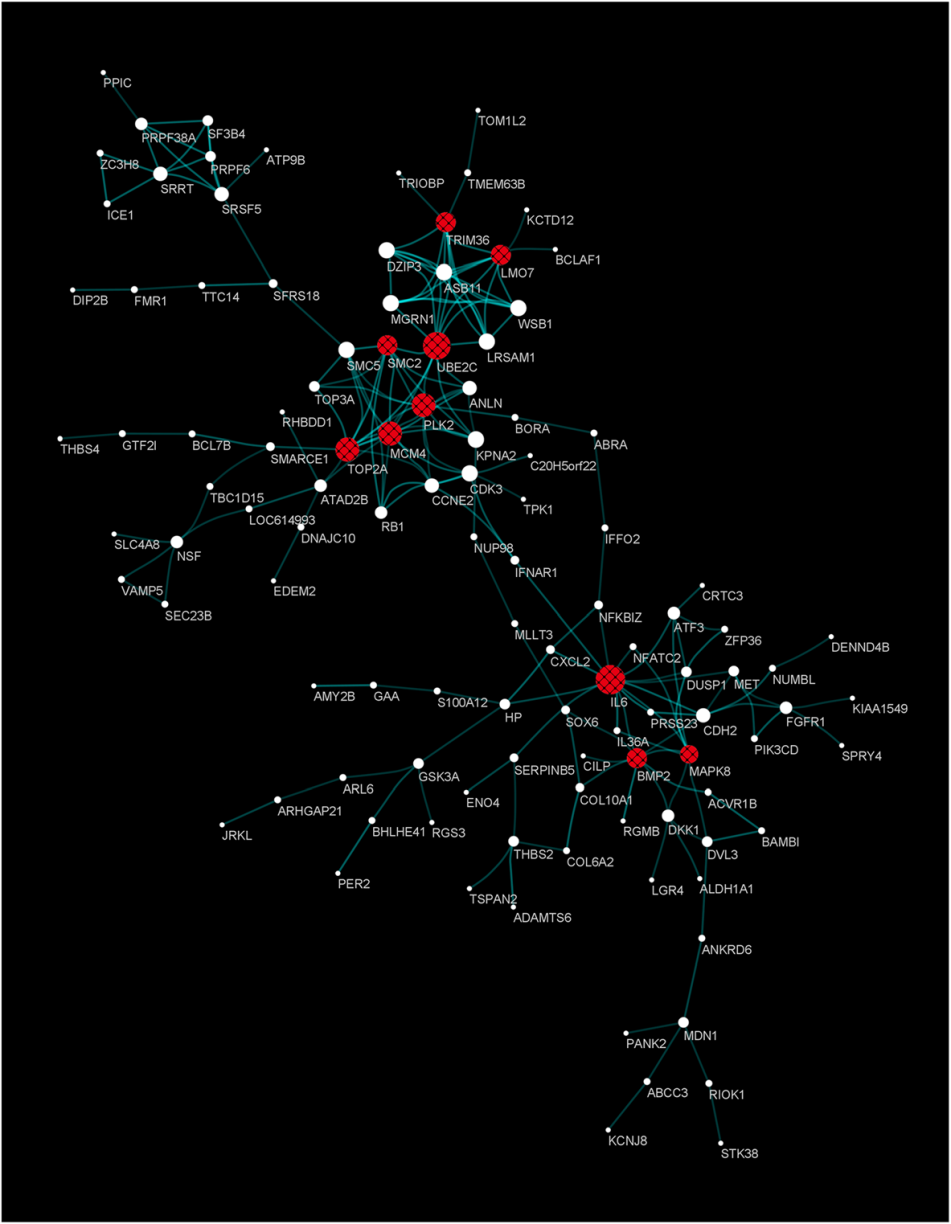
PPI network of all DEGs. Red nodes with mesh patterns represent hub genes analysed by the cytoHubba. Node sizes reflect the connection degree, the higher degree is, the larger node size is

The PPI network of all DEGs (Fig. 5), constructed through the STRING database, includes 113 nodes and 185 edges. Among them, DEGs, IL6, UBE2C, TOP2A, MCM4, PLK2, SMC2, BMP2, LMO7, TRIM36, and MAPK8 were screened as hub genes, according to their connection degrees (Table 4). IL6 displayed the highest degree (= 14), followed by UBE2C (= 13). The deletion of IL6 and UBE2C remarkably loosens the structure of the PPI network as it reduces the interaction between proteins. Therefore, IL6 and UBE2C are the core nodes of PPI, suggesting that IL6 and UBE2C play an important role in responding to dynamic compression.

**Table 4:** The top 10 hub genes

Rank	Gene symbol	Degree
1	IL6	14
2	UBE2C	13
3	TOP2A	11
3	MCM4	11
3	PLK2	11
6	SMC2	9
6	BMP2	9
6	LMO7	9
6	TRIM36	9
10	MAPK8	8

### Functional module analysis

**Fig. 6:**
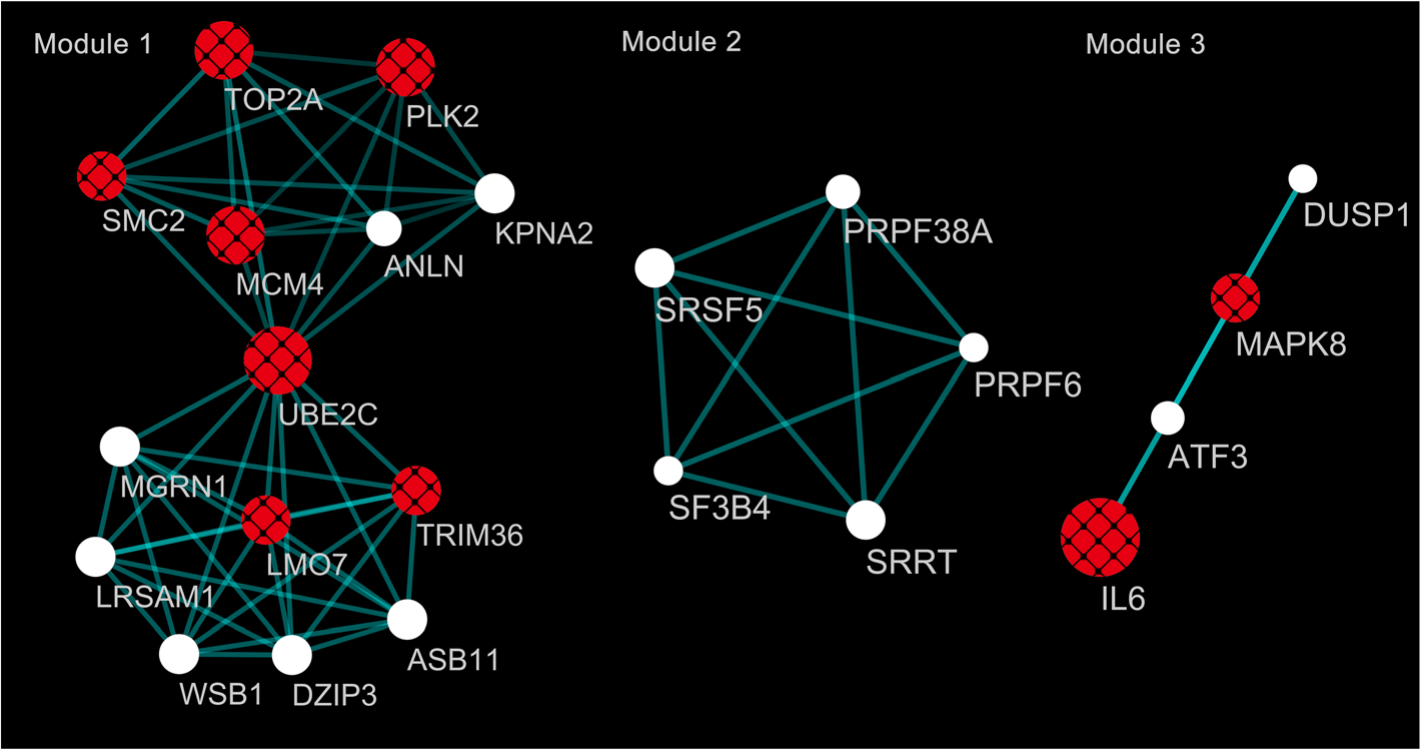
The top three most significant modules. Red nodes with mesh patterns represent hub genes analysed by the cytoHubba. Node sizes reflect the connection degree. The higher connection degree is, the larger node size is

The MCODE generated five sub-clusters, which reflect the high modularisation of a gene network. The top three amongst five modules contain nine of ten hub genes and are shown in Fig. 6. Module 1 consists of 14 nodes and 49 edges, and scores 7.54. Module 2 consists of 5 nodes and 10 edges, and scores 5.00. Module 3 consists of 4 nodes and 6 edges, and scores 4.00. As for annotation, this study focussed on Modules 1 and 3, which had the engagement of hub genes. Genes in Module 1 were mostly classified into GO terms of protein polyubiquitination, nuclear chromosome, and ATP binding, while genes in Module 3 were mainly classified into GO terms of defence responses to the virus, nucleus and cytokine activity (see Table 5). After screening and removing obvious irrelevant disease clusters, genes in Module 1 were mainly enriched through the ubiquitin-mediated proteolysis pathway, while the toll-like receptor signalling pathway, NOD-like receptor signalling pathway, cytosolic DNA-sensing pathway, and RIG-I-like receptor signalling pathway were identified for genes in Module 3 (see Table 6).

**Table 5:** Top 5 significantly enriched GO terms of module 1 and 3

Category	GO ID	Description	Gene Count	P-value
Module 1				
BP	GO:0000209	protein polyubiquitination	3	2.00 × 10^−3^
CC	GO:0000228	nuclear chromosome	2	9.21 × 10^−3^
MF	GO:0004842	ubiquitin-protein transferase activity	4	4.41 × 10^−4^
MF	GO:0008270	zinc ion binding	5	5.96 × 10^−3^
MF	GO:0061630	ubiquitin protein ligase activity	3	6.36 × 10^−3^
MF	GO:0005524	ATP binding	5	1.47 × 10^−2^
Module 3				
BP	GO:0071222	cellular response to lipopolysaccharide	2	2.01 × 10^−2^
BP	GO:0051607	defence response to virus	2	3.57 × 10^−2^
CC	GO:0005634	nucleus	3	1.59 × 10^−2^
CC	GO:0005615	extracellular space	2	2.24 × 10^−2^
MF	GO:0005125	cytokine activity	2	3.85 × 10^−2^

**Table 6:** Signalling pathway enrichment analysis of module 1 and 3

KEGG ID	Description	Gene Count	P-value	Gene list
Module 1				
bta04120	Ubiquitin mediated proteolysis	2	8.69 × 10^−2^	MGRN1, UBE2C
Module 3				
bta04620	Toll-like receptor signalling pathway	3	1.13 × 10^−3^	IL6, MAPK8
bta05142	Chagas disease (American trypanosomiasis)	3	1.33 × 10^−3^	IL6, MAPK8
bta05161	Hepatitis B	3	2.23 × 10^−3^	IL6, MAPK8
bta05164	Influenza A	3	3.04 × 10^−3^	IL6, MAPK8
bta05152	Tuberculosis	3	3.32 × 10^−3^	IL6, MAPK8
bta05168	Herpes simplex infection	3	3.66 × 10^−3^	IL6, MAPK8
bta04621	NOD-like receptor signalling pathway	2	2.68 × 10^−2^	IL6, MAPK8
bta04623	Cytosolic DNA-sensing pathway	2	3.19 × 10^−2^	IL6
bta04622	RIG-I-like receptor signalling pathway	2	3.97 × 10^−2^	IL6, MAPK8
bta05133	Pertussis	2	4.02 × 10^−2^	IL6, MAPK8
bta05132	Salmonella infection	2	4.33 × 10^−2^	IL6, MAPK8

## Discussion

Chondrocytes respond to mechanical stimuli through regulating gene expression, proliferation, and metabolic functions. However, little is known about the key genes, signalling pathways, and proteins. Chondrocytes have been considered a post-mitotic tissue with nearly no cellular turnover. They are surrounded by an extracellular matrix comprised of glycosaminoglycan (GAG) and collagen and are subjected to daily dynamic compression. During the in vitro culture, growth factors such as bone morphogenetic protein (BMP) and the TGF-β superfamily are indispensable for the chondrogenic differentiation of MSCs [16]. However, compared to native cartilage, cartilage induced by TGF-β alone showed inferior mechanical properties [17]. Dynamic compression was proved to stabilise the chondrogenic phenotype by inhibiting hypertrophy in the presence of TGF-β3 [18]. To sum up, dynamic compression is essential for inducing non-hypertrophic chondrogenesis of MSCs.

Furthermore, in Huang’s [15] original study, the results revealed that the timing of applying dynamic compression was important. The loading initiated soon after MSCbeing encapsulated into agarose, led to reduced mechanical properties. In contrast, loading initiated after chondrogenic induction and ECM elaboration in the presence of TGF-β3, enhanced the mechanical properties of MSC-seeded constructs. This may be attributed to different mechanotransduction pathways between differentiated and undifferentiated MSCs. Following a shift from the 2% agarose to a denser, cartilage-like construct, the stresses induction was higher. The microarray analysis of the original study showed that several genes from the MMP/TIMP family were significantly modulated. However, the original microarray analysis merely took the fold change of genes into consideration when evaluated the gene importance. This may lead to an inadequate revelation of actual hub genes, as the fold change of genes is not always reliable and proportional to the actual influence on cells. Considering the availability of original data, and the fact that dynamic loading with TGF-β3 is the proven condition that promoted a stable chondrogenic phenotype, this study was built up on one of Huang’s series experiments for further bioinformatics analysis. It explored how compressive stimuli influence the gene expression after chondrogenic induction using TGF-β3, to shed important insight on the mechanism behind. Although the study was initially intended to collect a series of datasets at different time points, the uploaded datasets involving mechanical loading were only available at the time point of day 42. As consequence, a possible loss of some gene information at the initial time point might become inevitable, nevertheless, the long-term gene modulation data at the ending time point was indispensable for analysis. New understanding resulting from the data excavation may contribute towards developing a better strategy to enhance chondrogenic efficiency, quality, and stability.

The high-throughput microarray technology combined with bioinformatics analysis has been widely used in providing new insight into gene expression changes and molecular mechanisms. In the present study, the GEO database was utilised to obtain microarray raw data. A total of 236 DEGs were identified between TGF-β3-induced and TGF-β3/dynamic-compression-induced MSCs, including 178 up-regulated genes and 58 down-regulated genes. After that, the DEGs were analysed by GO functional enrichment analysis and classified into three groups, which were subsequently further clustered, based on functions and signalling pathways.

The results of GO functional enrichment analysis showed that the DEGs were mainly enriched in the GO terms of inflammatory response, in utero embryonic development and negative regulation of angiogenesis. This conforms to previous studies showing that the inflammatory response was involved in chondrogenic regulation. Inflammatory factors have been recognised as an important driving force leading to cartilage breakdown, and their down-regulation is vital for constructing initial collagen networks. A previous animal study revealed that the three-day cyclic compression of 0.5 MPa at 0.5 Hz on bovine chondrocytes counteracted the cartilage degradation induced by IL-1 [19]. Therefore, dynamic loading is not only a stimulator for chondrogenesis, but also an anti-inflammatory factor against pro-inflammatory cytokines. In this study, there were two other GO terms – GO:0001701 (in utero embryonic development) and GO:0016525 (negative regulation of angiogenesis) – that were significantly abnormal between the TGF-β3-induced and TGF-β3/dynamic-compression-induced MSCs. This demonstrates that dynamic compression may affect the anatomical structure development of chondrogenesis. During the early embryogenesis and cartilage maturation, various mechanical stimuli in the microenvironment promote chondrogenesis and limb formation and are responsible for adult chondrocyte phenotype maintenance [20]. Generally, biomechanics has been widely regarded as a promoter of angiogenesis and osteogenesis [21, 22]. On the other hand, cartilage is an avascular system [3], however, the understanding regarding how cartilage maintains avascularity under a mechanical load is limited in the literature, and the underlying biomechanics have not yet been fully established. This study suggests that appropriate mechanical stimuli are vital for inducing less angiogenesis.

Moreover, KEGG pathways enrichment analysis was performed. Because the KEGG database integrates data on genomes, chemical molecules and biochemical systems, including pathways, drug, disease, gene sequences, and genomes, some irrelevant disease clusters might be unexpectedly enriched. These disease-related clusters were screened and removed from the results and discussion. The KEGG pathway enrichment of DEGs and module analysis showed that the PI3K-Akt signalling pathway, toll-like receptor signalling pathway and TNF signalling pathway were highly enriched. Studies have demonstrated that the activation of the PI3K-Akt pathway promotes the terminal differentiation of chondrocytes and inhibits the hypertrophic differentiation of chondrocytes [23, 24]. The toll-like receptors mainly use MyD88-dependent signalling to activate NF-κB to transcript pro-inflammatory cytokines. Moreover, the activation of the toll-like receptor-2 induces the chondrogenic differentiation of MSCs [25, 26]. On the other hand, the mechanical load may promote chondrogenesis by inhibiting the TNF signalling pathway to reduce cartilage degradation. Further investigation is desired to support these findings. In brief, the findings of identified GO terms and the KEGG pathways may provide a theoretical basis on how dynamic compression regulates chondrogenesis.

The PPI network was constructed to predict the connections of proteins encoded by DEGs. The top 10 hub genes were screened according to connection degree as follows: IL6, UBE2C, TOP2A, MCM4, PLK2, SMC2, BMP2, LMO7, TRIM36, and MAPK8. Nine of them functioned in two of the top three most significant modules, suggesting that these genes play a more important role in chondrogenesis and are enhanced by dynamic compression. The Modules 1 and 3 were extracted from the PPI network. UBE2C, TOP2A, MCM4, PLK2, SMC2 LMO7, and TRIM36 were contained in Module 1, which were mainly enriched in GO terms related to the cellular metabolic process. These genes have closed relationships with the cell cycle and proliferation, and some of them were found overexpressed in various tumours. Moreover, UBE2C [27], TOP2A [28] and MCM4 [29] were identified as DEGs in OA. However, to the best of our knowledge, there is as yet no study on how these genes function in MSCs differential regulation were enhanced by mechanical load. This needs further investigation.

It was reported that the downregulation of PLK2 inhibited the degree of inflammation of knee joint synovial tissue and inhibited the cartilage collagen destruction in rats [30]. In recent years, studies have revealed that the SMC family might regulate bone development via mitogenic signals and the Wnt pathway, which is a central pathway in the bone and cartilage differentiation [31]. However, little is known on the specific function of SMC2 in response to mechanical stimuli, which requires further study. LMO7 and TRIM36 are both cell cycle-related genes. The overexpression of TRIM36 decelerates the cell cycle and attenuates cell growth [32], however, their functions in chondrogenesis have not been identified. The IL6 and MAPK8 showed vital roles in Module 3, which GO terms were mainly enriched in response to stimuli and the immune system. The pro-inflammatory cytokine IL6 constitutes an important factor involved in inflammation, immunoregulation, haematopoiesis and tumorigenesis. Its function in chondrogenesis remains controversial. Some studies reported that IL6 inhibited the chondrogenic differentiation [33, 34], while others demonstrated that activating the IL6/STAT3 signalling pathway promoted homeostasis maintenance and cartilage regeneration [35]. It is speculated that mechanical stimulus within the appropriate range of intensity, duration, and frequency may function as a potent anti-inflammatory signal and impose a positive influence on chondrogenesis, while overloading and unloading may lead to cartilage degradation. MAPK8 belongs to the c-Jun N-terminal kinase (JNK), a family which is one of the three main categories of MAPK families. JNK activation represents a protective response to external stimuli. Mechanical stress may activate the JNK pathway by phosphorylating ERK1/2, p38 MAPK, and SAPK/ERK kinase-1 (SEK1), resulting in chondrogenic differentiation [36] and apoptosis regulation [37]. Collectively, the comprehensive findings from this study show that UBE2C, IL6, and MAPK8 may play more important roles in dynamic compression enhanced chondrogenesis, unlike the original study which suggested the MMP/TIMP family might be the key genes (15).

## Conclusions

This study analysed the gene expression profiles between TGF-β3-induced and TGF-β3/dynamic-compression-induced MSCs using a bioinformatics approach. 236 DEGs were found and annotated into GO terms and KEGG pathways, followed by constructing a PPI network and module mining. To our knowledge, this is the first time that genes, including UBE2C, IL6, and MAPK8, are identified to play a pivotal role in dynamic compression enhanced chondrogenesis via regulating proliferation, apoptosis and inflammatory response. Multiple signalling pathways, including the PI3K-Akt signalling pathway, toll-like receptor signalling pathway, TNF signalling pathway, and MAPK pathway, may be involved in sensation, transduction, and reaction of external mechanical stimuli. Although this is the first study giving a comprehensive genetic perspective on the interaction between mechanical stress and chondrogenesis, more experimental evidences are required to verify these findings. Further experimental studies are planned confirm these analysis results, which will be featured in the near future.

## Methods

### Microarray data information

The gene expression profiles of GSE18879 were downloaded from a public functional genomics data repository GEO database (https://www.ncbi.nlm.nih.gov/geo) [14] with the platform GPL2112 [Bovine] Affymetrix Bovine Genome Array (Affymetrix Inc., Santa Clara, CA, USA) [15]. This dataset includes negative control, TGF-β3-induced and TGF-β3/dynamic-compression-induced bovine bone marrow-derived MSCs specimens at three time points – day 3, 21 and 42 (repeated six times for each one). For specific groups, 10 ng/mL TGF-β3 was applied throughout 42 days, and the 10% strain dynamic compression at 1 Hz for 4 h daily began from day 21 onwards. Among them, the arrays of TGF-β3-induced and TGF-β3/dynamic-compression-induced specimens at day 42 were selected for analysis.

### Data pre-processing

The CEL format files of raw data were converted into probe expression matrix, then underwent background adjustment, quantile normalisation, and ssummarisation using the Robust Multichip Average (RMA) in the RMAExpress software (version 1.2.0) [38]. Then, a log2 transformation was performed on the gene expression levels when the expression matrix was exported. After that, the probe serial numbers were transformed into official gene symbols.

### Identification of DEGs

The up-regulated and down-regulated DEGs between TGF-β3-induced MSCs specimens and TGF-β3/dynamic-compression-induced MSCs specimens were identified through the Limma package on the NetworkAnalyst 3.0 web tool (https://www.networkanalyst.ca), which is a visual analytics platform for comprehensive gene expression profiling and meta-analysis [39]. Moreover, the *p*-value was corrected using the Benjamini-Hochberg test. Finally, the cut-off criterion of DEGs was set at the log2 fold change |log2FC| > 1.5 and adjusted as *P* < 0.05.

### GO and pathway enrichment analyses

The Database for Annotation, Visualisation, and Integrated Discovery (DAVID, https://david.ncifcrf.gov) is an online functional enrichment analysis web tool that provides systematic annotation information for the biological function of large-scale gene list [40, 41]. In this study, GO enrichment and KEGG pathway enrichment analyses of DEGs were performed using DAVID with a cut-off criterion of gene count > 2 and P < 0.05. The GO analysis comprises of biological processes (BP), cellular components (CC), and molecular functions (MF). Irrelevant disease clusters in the KEGG pathway enrichment analysis were screened and removed before analysis and discussion.

### PPI network construction

In order to understand the molecule mechanism and to study the interactions between dynamic compression and chondrogenesis, and between proteins encoded by DEGs and different proteins, the STRING (https://string-db.org) database [42] was utilised to recover the predicted associations between proteins encoded by DEGs and other proteins. The confidence score of > 0.4 was defined as significant. The results of the interaction data were then imported into the Cytoscape software (version 3.8.0) to visualise the PPI network. The degree distribution was established by counting the number of connections between different proteins in the network. The plug-in cytoHubba was utilised to screen the top 10 hub genes, ranked by degree.

### Functional module analysis

Another built-in APP Molecular Complex Detection (MCODE) was utilised to detect the dense functionally connected sub-clusters within the large PPI network. The parameters of network scoring and cluster finding were set as follows: degree cutoff = 2, node score cutoff = 0.2, k-core = 2, and max depth = 100. The top three sub-clusters identified by modularity analysis were then selected for GO and pathway enrichment analysis via DAVID (gene count > 2 and *P* < 0.05). Similarly, irrelevant disease clusters in the KEGG pathway enrichment analysis were screened and removed before analysis and discussion.

## Data Availability

The datasets generated and/or analysed during the current study are available in the GEO repository, https://www.ncbi.nlm.nih.gov/geo/query/acc.cgi?acc=GSE18879.

## Appendix

**Table 7:** Softwares and websites used in this paper

Software/website	Website adress
GEO database	https://www.ncbi.nlm.nih.gov/geo
Affymetrix Bovine Genome Array	https://www.affymetrix.com/support/technical/byproduct.affx?product = bovine
RMAExpress software (version 1.2.0)	https://rmaexpress.bmbolstad.com
NetworkAnalyst 3.0	https://www.networkanalyst.ca
DAVID	https://david.ncifcrf.gov
STRING database	https://string-db.org
Cytoscape software (version 3.8.0)	https://cytoscape.org

## Abbreviations

MSCs: Mesenchymal Stem Cells
GEO: Gene Expression Omnibus
TGF-β3: Transforming Growth Factor-Beta-3
DEGs: Differentially Expressed Genes
DAVID: Database for Annotation, Visualisation, and Integrated Discovery
GO: Gene Ontology
KEGG: Kyoto Encyclopedia of Genes and Genomes
PPI: Protein-Protein Interaction
FAK: Focal Adhesion Kinase
MAPK: Mitogen-Activated Protein Kinase
GEO: Gene Expression Omnibus.
GAG: Glycosaminoglycan.
BMP: Bone Morphogenetic Protein.
JNK: c-Jun N-terminal Kinase.
SEK1: SAPK/ERK Kinase-1.
YAP: Yes-Associated Protein.
TAZ: Transcriptional Coactivator with PDZ-binding Domain.
PUU: Poly (urea-urethane).
3D-TIPS: 3-Dimension Printing-guided Thermally Induced Phase Separation.
